# Somatostatin Receptors in Merkel-Cell Carcinoma: A Therapeutic Opportunity Using Somatostatin Analog Alone or in Association With Checkpoint Inhibitors Immunotherapy. A Case Report

**DOI:** 10.3389/fonc.2020.01073

**Published:** 2020-07-07

**Authors:** Michele Guida, Alessandro D'Alò, Anita Mangia, Federica Di Pinto, Margherita Sonnessa, Anna Albano, Angela Sciacovelli, Artor Niccoli Asabella, Livia Fucci

**Affiliations:** ^1^Department of Medical Oncology, IRCCS Istituto Tumori “Giovanni Paolo II”, Bari, Italy; ^2^Functional Biomorphology Laboratory, IRCCS Istituto Tumori “Giovanni Paolo II”, Bari, Italy; ^3^Department of Pathology, Research Hospital, National Institute of Gastroenterology “S. De Bellis”, Castellana Grotte, Italy; ^4^Department of Pathology, IRCCS Istituto Tumori “Giovanni Paolo II”, Bari, Italy; ^5^Nuclear Medicine Unit, Department of Interdisciplinary Medicine, University of Bari Aldo Moro, Bari, Italy

**Keywords:** immunothearpy, somatostatin analog, Merkel cell carcinoma (MCC), Merkel carcinoma, somatostatin—receptor

## Abstract

**Background:** Merkel-cell carcinoma (MCC) is a rare, highly aggressive skin cancer typically involving elderly people. Surgery is usually the first treatment for primary tumor. In adjuvant setting, radiotherapy is effective in reducing local recurrence and in improving overall survival. Regarding advanced disease, systemic chemotherapy ended up disappointing results whereas antiPD1/antiPD-L1 immunotherapy recently gave relevant clinical benefits. Interestingly, about the half of MCC patients expresses high somatostatin receptors (SRs) to possibly represent a target for the therapeutic use of somatostatin analogs (SSAs). Nevertheless, SSAs have been little studied in MCC and cases treated with SSAs in association with checkpoint inhibitor immunotherapy have not been published yet.

**Case Report:** We report the case of a 73-year-old man affected by metastatic MCC of right arm previously treated with surgery and adjuvant radio and chemotherapy. Three years later the patient presented loco-regional relapse involving lateral-cervical, mediastinal, and submandibular lymph nodes with high value of chromogranin A and neuron specific enolase. Due to the high expression of SRs at octreoscan and immunoistochemistry, patient started octreotide 30 mg i.m. every 28 days with a good control of disease for about 2 years. A widespread progression of disease was reported afterwards. The patient started the antiPD-L1 avelumab immunotherapy, only recently available in Italy, while still taking SSA. The patient showed an impressive regression of the disease after only four cycles of avelumab until complete remission.

**Conclusions:** SSA could be a valid therapeutic option in patients with MCC with high SR expression. When combined with PD-1/PD-L1 immune-checkpoint inhibition, SSA is likely to enhance antiproliferative activity. Our case report provides the rationale to conduct a prospective trial and translational research to verify the efficacy and safety of combined SSA and checkpoint inhibitors for advanced MCC.

## Introduction

Merkel-cell carcinoma (MCC) is a rare but highly aggressive skin cancer typically involving elderly people, although it has been also described in young adult and exceptionally in childhood (1).

Factors involved in the pathogenesis of MCC included age over 65 years, ultraviolet radiation exposure, immunosuppression, and infection by Merkel cell polyomavirus (MCPV) which is detected in almost 80% of MCC cases. Other primary cancers seem to increase the risk of MCC incidence, especially prior multiple myeloma, chronic lymphocytic leukemia, and malignant melanoma (2). Ultraviolet light exposure has been reported to induce an extremely high genome mutation rate, whereas MCPV-infected Merkel carcinoma cells show low rates of genome mutation (3).

MCC typically tends to grow locally, but soon it spreads to the locoregional lymph nodes and than through the blood circulation to distant organs, particularly to liver, lung, brain, and to bone (4).

Therapeutic management of MCC is controversial. Early diagnosis and adequate treatment of primary MCC are important prognostic factors. Surgery and radiotherapy are usually chosen in localized forms. Adjuvant radiotherapy showed effective in reducing the local recurrence and in increasing the overall survival (5).

Systemic chemotherapy has been used to treat advanced disease with disappointing results. First-line chemotherapy with platinum-based regimens produced high response rates of about 50–60%. The main therapeutic regimens included cis-platinum or carboplatin in association with etoposide or ifosfamide or anthracyclines. CAV regimen (cyclophosphamide + Adriamycin + Vincristine) was used in patients unfit for platinum-based regimens. Unfortunately, response duration was only a few months with PFS of 3–4 months. Moreover, treatment was associated to significant toxicity (6, 7).

Checkpoints inhibitors antiPD1/antiPD-L1 (Programmed Death Ligand1) immunotherapy have been recently investigated in the metastatic setting and positive results were reported (8–10). The antiPD-L1 avelumab was first tested in a multicentre phase 2 trial involving 88 patients with stage IV chemotherapy-refractory MCC. The response rate was 31.8%, including eight complete responses and 20 partial responses (8). Based on these results, the U.S. Food and Drug Administration granted accelerated approval of the antiPD-L1 avelumab to treat adults and children above 12 years with metastatic MCC.

Other two antiPD-1 antibodies have also been investigated in advanced MCC. Pembrolizumab was tested as first-line treatment in advanced MCC (9) whereas nivolumab was proposed as neoadjuvant therapy in patients with resectable MCC (10). In both studies, an objective response rate over 50% was reported. Of note, responses were observed in both patients with virus-positive tumors and those with virus-negative tumors (9–11).

Due to these new therapeutic options, chemotherapy is now indicated just for patients who are not candidates for immunotherapy or after immunotherapy failure.

About half of MCC expresses highly somatostatin receptors (SRs) that could represent a potential target for both imaging and treatment purposes (12). Somatostatin analogs (SSAs) have been used in the past with palliative intent for functioning neuro-endocrine tumors and remarkable results were reported. More recently, direct anti-proliferative effects of SSAs have also been demonstrated in neuroendocrine neoplasms (13). The use of SSA in MCC has been little studied (14) and cases of MCC treated with SSA in combination with checkpoint inhibitor immunotherapy have not been published yet.

We report for the first time the case of a metastatic MCC successfully treated first with SSA and then, when disease progressed, with SSA plus anti PD-L1 avelumab.

## Case Report

**FIGURE 1:**
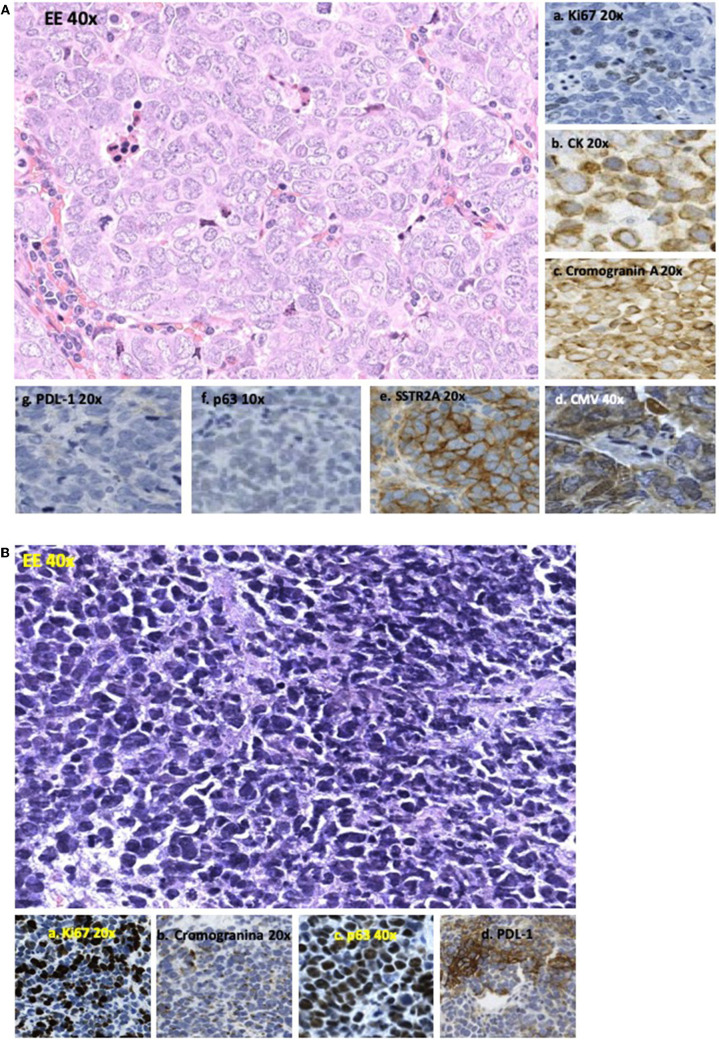
**(A)** Sample of lymph node metastasis performed before SSA therapy showing an epithelioid feature of cells with “salt and pepper” nuclei (EE40x). Immunohistochemistry showed (a) positivity for Ki67 in 25% of cells (20×); (b) positivity for CK20 with nuclear dot (20×); (c) positivity for chromogranin A with nuclear dot (20×); (d) nuclear positivity for poliomavirus Merkel cells carcinoma associated/CMV (40×); (e) diffuse and strong membranous positivity for somatostatin receptor 2A (SSTR2A) (20×); (f) negativity for p63 (10×); and (g) for PDL-1 (20×). **(B)** Sample of lymph node metastasis performed after disease progression to SSA therapy showing cells having smaller size than those of pre-treatment and a round shape with dark nuclei (40×). Immunohistochemistry showed (a) Ki67 positivity in 85% of cells (20×); (b) reduction of positivity for chromogranin (20×); (c) diffuse nuclear positivity for p63 (40×); (d) strong positivity for PDL-1 in 35% of neoplastic cells (10×).

A 73-year-old man affected by metastatic MCC on the right arm treated with surgery and adjuvant radio and chemotherapy came to our observation in December 2015. At the diagnosis, immunohistochemistry reported a Ki67 of 25% and a positivity for synaptophysin, chromogranin A, CK20, nuclear polyomavirus marker, and SSTR2A, whereas SSTR5A, PD-L1, and p63 (a transcription factor often associated with worse prognosis) were negative (Figure 1A). The tumor infiltrating lymphocytes (TILs) in the tumor microenvironment were moderate as was the infiltration of intra-tumor lymphocytes.

Three years later the patient presented relapse involving loco-regional lymph nodes and the right lung with high value of chromogranin A (CgA) and neuron specific enolase (NSE).

**FIGURE 2:**
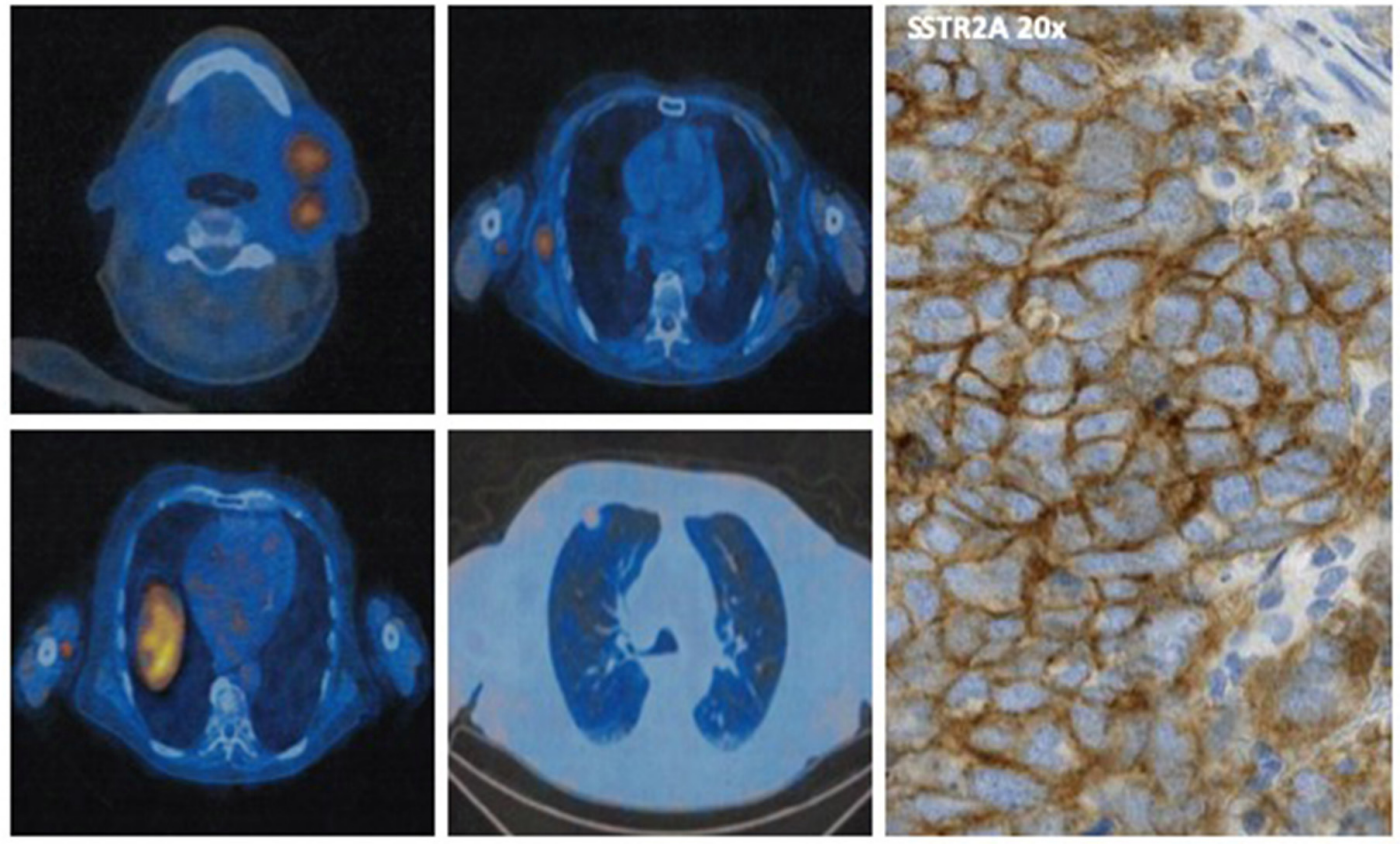
Octreoscan showing high uptake of 99mTc-EDDA/HYNIC-TOC in the right axillary and thoracic wall, in the left sub-mandibular and latero-cervical region, and at right superior pulmonary lobe in the sub-pleurical region. On the right is shown the membranous diffuse expression of SSTR2A on immunohistochemistry.

Due to the high expression of SRs at octreoscan with 99mTc-EDDA/HYNIC-TOC (Figure 2) and at immunohistochemistry (Figure 1A), the patient started the SSA octreotide at the dose of 30 mg intramuscularly every 28 days obtaining a progressive decrease of serum markers and a partial regression of disease lasting over 2 years. After this long period, the patient presented disease progression in all known sites and new lesions at lung middle lobe and left adrenal gland. At this time, circulating CgA remained under normal value whereas NSE increased rapidly (**Figure 4**). A new lymph node biopsy was performed revealing a profound changing in the morphology of the neoplastic cells. Immunohistochemistry also showed a Ki67 of 85% compared to 25% of the first biopsy, whereas p63 and PD-L1 became strongly positive (Figure 1B). At this time, peritumoral TILs were very scarce and completely absent the intra-tumor lymphocytes component.

**FIGURE 3:**
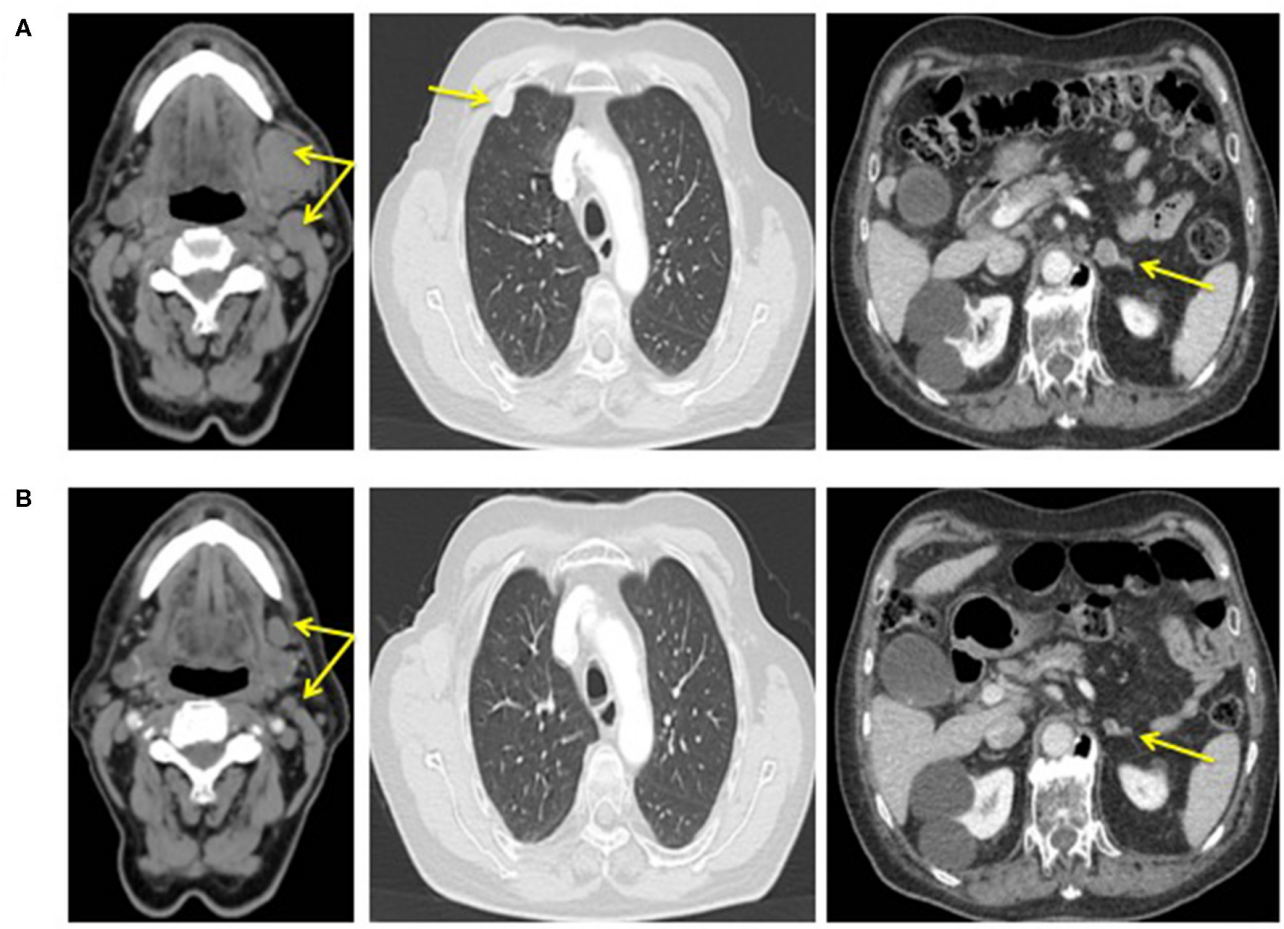
CT scan performed before therapy with the association octreotide plus avelumab **(A)** and after 10 months of therapy **(B)**.

**FIGURE 4:**
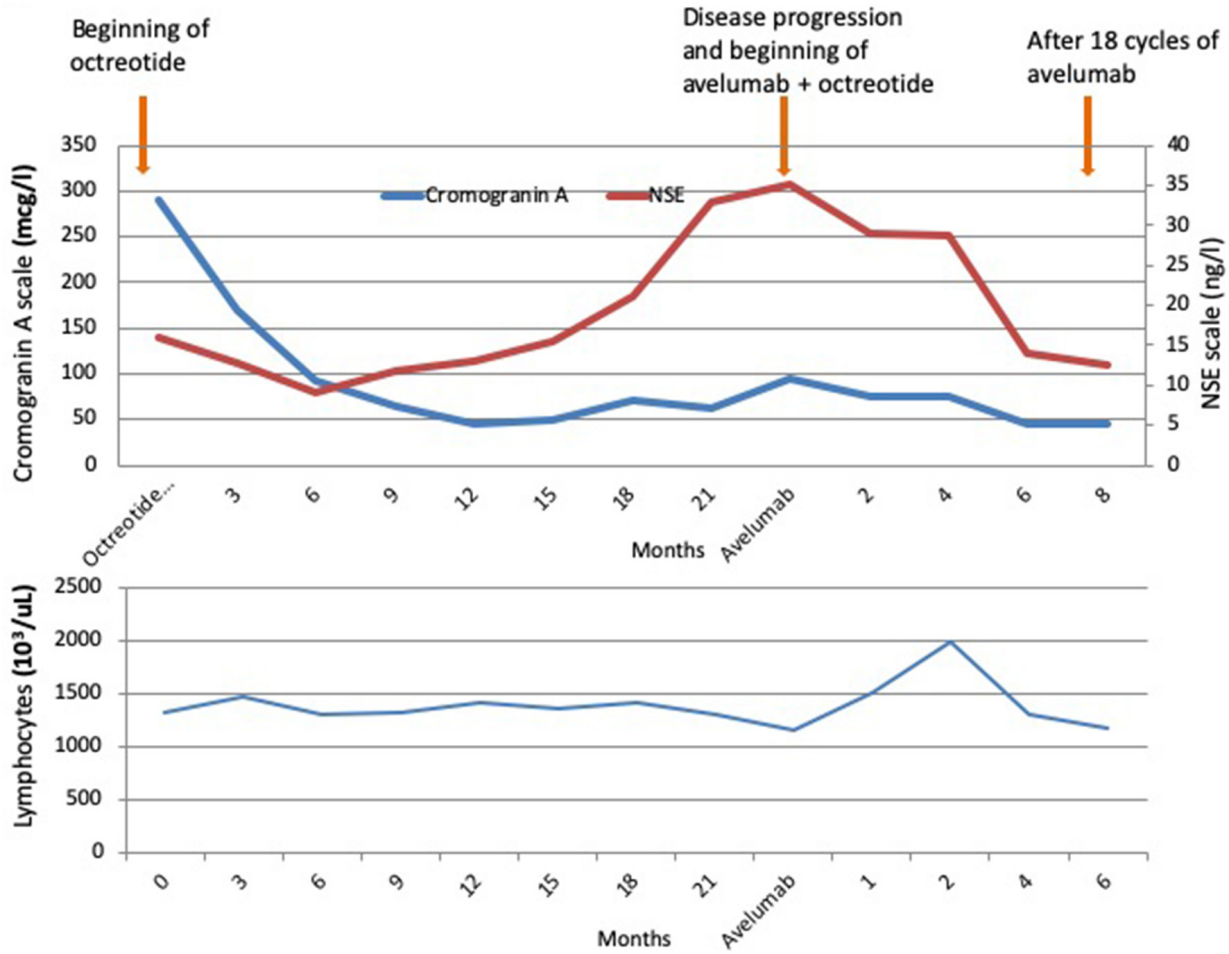
Behavior of chromogranin A (CgA), Neuron-Specific Enolase (NSE), and peripheral lymphocytes count during octreotide and then during the association of octreotide plus avelumab.

Considering the disease progression and the recent availability in Italy of the antiPD-L1 avelumab, the patient started this drug at the dose of 10 mg/kg intravenously every 2 weeks continuing octreotide at the same dose and timing. After four administrations of avelumab, an impressive response was observed until achieving a complete remission (Figures 3A,B). Also circulating NSE level progressively decreased until normal value (Figure 4).

Peripheral lymphocytes remained quite under normal value during all SSA therapy period without significant variations. Nevertheless, when avelumab was started, a sudden and rapid increase just after the first administration of avelumab was noted reaching the maximum value at the second month of therapy. Then, lymphocytes progressively decreased until pre-treatment value (Figure 4).

Treatment was well tolerated by the patient with only mild hyposthenia reported.

At present, after 17 months of therapy, the patient is in good clinical status with complete remission of disease. He is continuing avelumab in combination with octreotide without remarkable side effects.

## Discussion

Avelumab was the first-ever drug approved in advanced MCC after showing meaningful efficacy. In the second line setting after chemotherapy, the response rate reaches 33% with responses occurring on average after about 6 weeks of therapy. Early objective response to avelumab was associated with significant improved overall survival. Moreover, in patients who responded, about two-third remained relapse free after 2 years with half of patients and one-third of patients still alive at 1 and 2 years, respectively. When used in a first-line setting, PD-1/PD-L1 inhibitors including avelumab, pembrolizumab, and nivolumab are even more promising as objective responses were observed in ~50–70% of patients with a 2-year survival rate of about 70%. Due to these results, PD-1/PD-L1 inhibitors are considered the standard of care in advanced MCC and are currently being investigated in the adjuvant and neoadjuvant settings (9–11).

Somatostatin is a hormone with multiple actions such as endocrine, paracrine, and antiproliferative. The direct antiproliferative effects of somatostatin are mediated by its high affinity for somatostatin specific receptors (SRs) present on neuroendocrine neoplasms (15–17). Five sub-types of specific membrane receptors have been found in a lot of normal tissues as brain and leptomeningeal tissue, anterior pituitary gland, endocrine and exocrine pancreas, gastrointestinal mucosa, immune system cells (16, 17). Of note is that each type of tumor shows different sub-types of SRs and the amplitude of the antiproliferative activity of somatostatin analogs (SSAs) seems to correlate with the number of receptors present on the cellular surface (16, 17).

SSTR2A and 5 have been recently reported to be expressed in MCC in about 60 and 45%, respectively (12). Overall, at least one SR was expressed in 76.5% of cases and no association was found either with the severity of the disease, nor with clinical features, proliferative index of Ki 67, relapse-free survival, and overall survival. SRs expression between the primary skin tumor and the corresponding metastases was consistent in 43 and 86% for SRs 2A and SRs 5, respectively. The expression of SSTR2A but not SRs 5 was finally associated with MCPyV status (12).

Considering the expression of these receptors, this tumor may be candidate for SSAs therapy. To our knowledge, the use of SSAs in MCC has been little investigated with only three cases published in English literature and disappointing results were reported (14). No cases treated with SSAs plus checkpoints inhibitors have been published until now.

We obtained a good control of the disease for about 2 years using octreotide alone. Of note, when the patient presented a widespread progression of the disease, a radical change in terms of cell morphology and higher proliferative index were noted, whereas p63 and PD-L1 became strongly positive (Figure 1B). The behaviors of tumor markers also confirmed the biological switch of disease with CgA remaining within normal values and NSE slowly increased (Figure 4).

We obtained an impressive regression of the disease with avelumab in association with octreotide until complete remission lasting over 10 months without relevant side effects with only mild hyposthenia reported by our patient. Data from literature regarding the toxic profile of avelumab reported that this drug is associated with a very good toxicity profile. No treatment-related grade 4 adverse events or treatment-related deaths were reported. Moreover, grade 3 treatment-related adverse events occurred in only 6% of patients with lymphopenia, blood creatine phosphokinase increase, aminotransferase increase, and blood cholesterol increase being more common side effects. Finally, serious treatment-related adverse events included enterocolitis, infusion-related reaction, aminotransferases increased, chondrocalcinosis, synovitis, and interstitial nephritis. All side effects were effectively addressed with prompt recognition and appropriate management (8).

Interestingly, peripheral lymphocytes which showed no significant variation during SSA therapy, increased significantly after the first administrations of avelumab with the maximum value at the second month of therapy and then progressively decreased.

Although considerable therapeutic progress has been made in the MCC with the arrival of checkpoint inhibitors, about 50% of patients with advanced disease do not respond to immunotherapy. Unfortunately, no clear predictors of response are available yet. Regarding PD-L1, its prognostic value is controversial as well as its predictive role of response to checkpoint inhibitors. Although more remarkable responses have been reported in cancer overexpressing PD-L1 levels regardless of histology, as the case of our patient, even patients with low PD-L1 levels can have an important clinical benefit with immunotherapy. Our patient showed high expression of PD-L1 on tumoral cells before starting therapy with avelumab. Therefore, using PD-L1 as predictive factor is questionable for its weak and unreliable capability to discriminate responder from no-responder patients. Thus, PD-L1 could constitute one of the partners when associated with other finer and more specific parameters such as mutational load, tumor neoantigen burden, cytotoxic activity, and IFN gamma signature. It is urgent to identify new predictive factors of response to immunotherapy. It is thought that better understanding of tumor microenvironment and use of combined biomarkers are necessary to better identify patients who will benefit from PD-1/PD-L1 checkpoint blockade therapy. Moreover, new immunotherapeutic strategies are now being investigated both alone or in combinations to enhance the immune antitumoral response against (18, 19).

Our case demonstrates that SSA represents a valid therapeutic option in MCC patients expressing high SRs levels. When combined with check point inhibitors immunotherapy, SSA can safely enhance the anti-proliferative activity of immunotherapy with final strengthen results. Thus, it provides a rationale to conduct a prospective trial with an adequately powered sample to test the efficacy and safety of this combination and to optimize the schedule and timing of the two drugs. Furthermore, translational research is also recommended to better characterize the potential immune activation properties and the synergistic activity of SSA and to verify the predictive value of SRs to response to this therapeutic combination.

## Data Availability Statement

All datasets generated for this study are included in the article/supplementary material.

## Ethics Statement

Written informed consent was obtained from the patient for the publication of this case report.

## Author Contributions

All authors listed have made a substantial, direct and intellectual contribution to the work, and approved it for publication.

## Conflict of Interest

The authors declare that the research was conducted in the absence of any commercial or financial relationships that could be construed as a potential conflict of interest.
